# Neural correlates of decision making and executive function in suicidal thoughts and behaviors

**DOI:** 10.3389/fpsyt.2025.1676986

**Published:** 2025-12-10

**Authors:** Fangfang Tian, Lu Zhou, Xiuli Wang, Neil Roberts, Hua Pang

**Affiliations:** 1 Department of Nuclear Medicine, The First Affiliated Hospital of Chongqing Medical University, Chongqing, China; 2 Department of Nuclear Medicine, Tangshan People’s Hospital, Tangshan, China; 3 Department of Clinical Psychology, The Fourth People’s Hospital of Chengdu, Chengdu, China; 4 Centre for Reproductive Health (CRH), Institute for Regeneration and Repair (IRR), University of Edinburgh, Edinburgh, United Kingdom

**Keywords:** suicide thoughts and behaviors, decision-making, executive function, fMRI, meta-analysis

## Abstract

**Background:**

To investigate the neural alterations associated with decision-making (DM) and/or executive function (EF) in psychiatric patients with suicidal thoughts and behaviors (STBs).

**Methods:**

Systematic searches for relevant publications were performed using the PubMed, ScienceDirect and Web of Science databases. A method of quantitative coordinate-based meta-analysis, known as anisotropic effect size version of seed-based d mapping (ASE-SDM), was used to locate brain regions displaying anomalous activations in patients with STBs compared to patient controls (PCs) based on DM and EF tasks, separately. Moreover, we used multimodal analysis to investigate the neural correlates of DM and EF tasks in the brain. Additionally, sensitivity analysis was conducted to assess the robustness of the results, and publication bias was evaluated to ensure the reliability of the findings.

**Results:**

The results pertaining to the DM tasks revealed significant hyper-activations of the left anterior cingulate cortex (BA 24, p = 0.000371) and right insula (p = 0.000640), together with hypo-activations of left insula (p = 0.000387) and left hippocampus (p = 0.0000619) in patients with STB compared to PCs. During the EF tasks, patients with STB only showed hyper-activations in the left anterior cingulate cortex (BA 24, p = 0.00121) and left precentral gyrus (p = 0.00391) compared to PCs. The multimodal analysis elucidates the significance of the cingulate cortex in both DM and EF processes.

**Conclusions:**

Our results suggest that dysregulated neural activity of the ACC is a key mechanism contributing to suicidal risk, with DM abnormalities playing a more central role than EF deficits. These findings highlight potential targets for interventions, such as cognitive-behavioral therapies focusing on DM and impulsivity, or targeting the shared brain region in the left ACC, which could reduce suicidal behavior. Addressing emotional regulation through mindfulness-based therapies may also be beneficial. Future research should validate these interventions and explore their long-term efficacy. This study has been registered in PROSPERO (number CRD42022340922).

## 1 Introduction

Suicidal thoughts and behaviors (STBs) are not only a personal mental health issue but also a significant public and social health challenge that impacts global economic and social development (1). Despite a growing number of studies to understand the risk factors and mechanisms underlying suicidal behavior the rates of suicide remain high.

Studies of populations at high risk for suicide have showed that STB is linked to impairments in decision making (DM) and executive function (EF) (2), which are two related but different cognitive processes. DM primarily encompasses the prediction, risk assessment, and value judgment of future outcomes, whereas EF pertains to capacity to direct behavior and suppress irrelevant or automatic responses. A substantial body of literature exists relating to study of the biological and psychological factors associated with impaired DM and EF in STB (3, 4). It has been suggested that deficiencies in EF are a common pathway leading to suicidal behavior (5), and deterioration of EF is the principal factor contributing to impaired DM in individuals with depression, significantly influencing the DM process through aspects such as inhibitory control and cognitive flexibility (6). Conversely, some researchers have suggested that impaired DM is the principal factor associated with susceptibility to suicide in individuals (7). The present study has been conceived to shed light on the question of whether deficits in DM or EF have greater influence on STB.

In recent years, numerous studies have investigated the association between suicidal behavior and neurocognitive factors. A meta-analysis conducted by Escobar et al. (8) revealed that, despite absence of significant differences in response time, individuals with a history of suicide attempts exhibited significant deficits in the performance of EF tasks. With regard to individual studies, Gifuni et al. (9) found evidence of deficits in both DM and EF in adolescents who exhibited suicidal behavior, Perrain et al. (10) reported that particularly individuals who have resorted to violent methods of attempted suicide, exhibit a propensity for making higher-risk choices in DM tasks, and Sastre-Buades et al. (7) reported that individuals with suicidal tendencies generally demonstrate poorer performance in DM tasks. Collectively, these studies underscore the critical role of impairments in both DM and EF in suicidal behavior, however whether one is more predominant than the other remains to be elucidated. Functional magnetic resonance imaging (fMRI) is an indispensable advanced neuroimaging technology for assessing cognitive impairment and prefrontal cortex (PFC), especially medial prefrontal cortex (mPFC), consistently emerges as the brain region that is most frequently activated in the performance of DM and EF tasks. Interestingly, alterations in activation of PFC (11, 12), orbitofrontal cortex and amygdala (13) have been reported in individuals with STB during performance of DM tasks, whereas alterations of PFC, (14) basal ganglia and anterior cingulate cortex (ACC) have been reported in individuals with STB during performance of EF tasks (15, 16). While these findings contribute significantly to understanding the neural underpinnings of DM and EF in individuals with STB, results from individual studies often rely on data from small samples which may lead to inconsistencies and low reliability (17). Consequently, in the present study a quantitative meta-analysis of all previous fMRI studies of DM and EF in patients with STB has been performed. Importantly, studies that derived brain areas based on *a priori* hypothesis have been excluded to avoid potential bias. Additionally, sensitivity and meta-regression analyses have been performed to confirm the accuracy of the findings that are reported.

## 2 Methods

### 2.1 Search strategies

A systematic and comprehensive search was conducted in the PubMed, ScienceDirect, and Web of Science databases on Sep 9, 2024 to identify fMRI studies of patients with STB during DM and/or EF task. The following search terms were used: (“suicide” OR “suicidal” OR “suicidality”) AND (“mental processes” OR neuropsychology OR decision-making OR “decision making” OR reward OR “executive function*” OR “cognitive control” OR inhibition OR updating OR shifting OR “working memory”) AND (fMRI OR “functional magnetic resonance imaging”). There was no restriction on the publication date and only original articles published in English were considered, and the reference lists of relevant review papers (18–20) were checked for additional publications related to the topic. The Preferred Reporting Items for Systematic Reviews and Meta-Analyses (PRISMA) guidelines (http://Prisma-statement.org) have been adhered to throughout the present study (**Supplementary Table S1**), and the study underwent an assessment by PROSPERO (CRD42022340922).

### 2.2 Selection criteria

Studies were included if they met the following criteria: (i) the patient group included individuals with a history of suicidal ideation and/or suicide attempt (SA), collectively referred to as STB, with no limitation on age, sex and psychiatric comorbidities, (ii) the control group was defined as patients with the same psychiatric disorder as the patient group but without STB in their lifetime, (iii) task-based fMRI had been performed to investigate DM and/or EF, (iv) the control task encompassed all elements of the associated task condition, except for the specific aspect of interest, (v) studies were based on whole-brain analysis, (vi) brain areas were reported as peak coordinates in stereotactic space. Reviews, abstracts, theses, case reports, editorials, letters, and conference proceedings were excluded in the analysis.

### 2.3 Review selection

Screening of the studies which the search had identified for potential inclusion in the meta-analysis was carried out by TFF and ZL using EndNote X8. Firstly, duplicates were removed. Secondly, unwanted types of literature were excluded by scrutinizing keywords in the titles and abstracts. Thirdly, a thorough examination of the titles and abstracts of the studies to potentially be included was performed to assess relevance. During this process, the full texts of relevant articles was read, with a focus on the Methods section and particular attention paid to the participants that were recruited, MRI that was performed, fMRI tasks used, and statistical analyses that was performed. Studies that met the following criteria were categorized as “possibly included”: (i) patients at a high risk of suicide, including those with STB, (ii) all participants completed a DM and/or EF fMRI task and (iii) regions of brain activation were reported. Studies categorized as “possibly included” underwent further detailed screening until a final decision could be made. For example, if the coordinates of peak activation were not reported the primary author was contacted to request whether this information could be provided.

### 2.4 Data extract and quality assessment

The following information was compiled for all the studies that were selected for inclusion in the meta-analysis: first author, year of publication, cohort size, demographics (including sex, age, type of suicide and psychiatric diagnosis), task information (including type of task and task performance), imaging parameters (such as magnetic field strength, stereotactic template use and name of analysis software), medication and statistical threshold. Prior to the meta-analysis, the reported peak coordinates of activity and their corresponding t-values were extracted into a text file. During the extraction process, the following considerations were taken into account. Firstly, peak coordinates (x, y, z) reported in Talairach space were converted to MNI space using the ‘tal2icbm_fsl’ transform (http://www.brainmap.org/icbm2tal/), as described by Lancaster et al. (21). Secondly, in cases where statistical significance was reported as a z-value this was converted to a *t*-value using the Statistics Converter (http://www.sdmproject.com/utilities/?show=Statistics). Thirdly, if no *t* or *z*-value was reported, a ‘*p*’ or ‘*n*’ (indicating a positive or negative direction of activation, respectively) was chosen as a substitute (22). Finally, once the text files containing the extracted data from all studies had been prepared, a table was created in which each line represents a study and includes the name of the first author, participant sample size and t*t* statistic. This extraction process was performed by ZL and was subsequently reviewed by TFF.

To assess the quality of individual studies, a checklist based on common elements from existing criteria for assessing psychoradiology studies was used. The checklist, which was tailored to the objectives of the present study, consisted of 10 items organized into three categories, namely participant inclusion, image acquisition and analysis, and results reporting (**Supplementary Table S2**). The checklist was independently scored by two investigators (TFF and ZL) and any discrepancies were resolved by consensus following discussion.

### 2.5 Main meta-analysis

#### 2.5.1 Meta-analysis of DM tasks

A coordinate-based meta-analysis based on DM tasks was performed using version 5.15 of the anisotropic effect-size signed differential mapping software (AES-SDM, http://www.sdmproject.com). This software allows for the reconstruction of a 3D statistical image of the regions of brain activation that can be used for group-level analysis. Details of the methods have been described elsewhere (22), and the main steps of the analytic procedure to create the statistical image are as follows. Firstly, peak coordinates were convolved with an anisotropic Gaussian kernel with full width at half maximum (FWHM) of 20 mm, and voxel size of 2 mm within a full brain mask. The choice of full anisotropy (i.e. anisotropy = 1) and FWHM of 20 mm align with standard SDM guidelines and recommendations made by Radua et al. (23) Secondly, the AES-SDM generated Hedges’ effect sizes and their associated variances were computed based on the peak coordinates and t statistics extracted from the original studies by employing standard formulas (22). Thirdly, the peak effect size and Gaussian kernel were utilized to estimate the lower and upper bounds of possible effect sizes for each study. Finally, the most likely effect size and its standard error were estimated for all voxels in the statistical image.

#### 2.5.2 Meta-analysis of EF tasks

Brain activation differences of EF task between patients with STB and PCs were also analyzed with a similar procedure with DM meta-analysis.

### 2.6 Multimodal overlapping of DM and EF tasks in STB

In this study, to clarify whether DM and EF have the same activation pattern in brain regions in patients with STB. Therefore, we overlapped DM and EF tasks altered regions to examine convergence in results from different task types using the “multimodal” analysis of SDM software with the default option (i.e., to find the regions presenting differences both at the DM and EF task).

### 2.7 Sensitivity and heterogeneity analyses

A Jack-knife sensitivity analysis, in which one dataset was excluded at a time to determine whether the results remained significant, was performed to assess the reliability of the results. Lastly, the Egger test (24), as implemented in SDM, was used to assess the asymmetry of funnel plots so as to detect any potential publication bias for DM and EF studies, separately.

### 2.8 Meta-regression and subgroup analyses

Furthermore, meta-regression analyses were performed to examine the potential effects of sex, age and sample size across studies, with these variables serving as predictors and using a threshold of p < 0.0005 for DM and EF studies, separately.

We conducted an additional subgroup analysis of studies of SA and suicide ideation respectively.

## 3 Results

### 3.1 Descriptive characteristics and quality assessment of included studies

**Figure 1:**
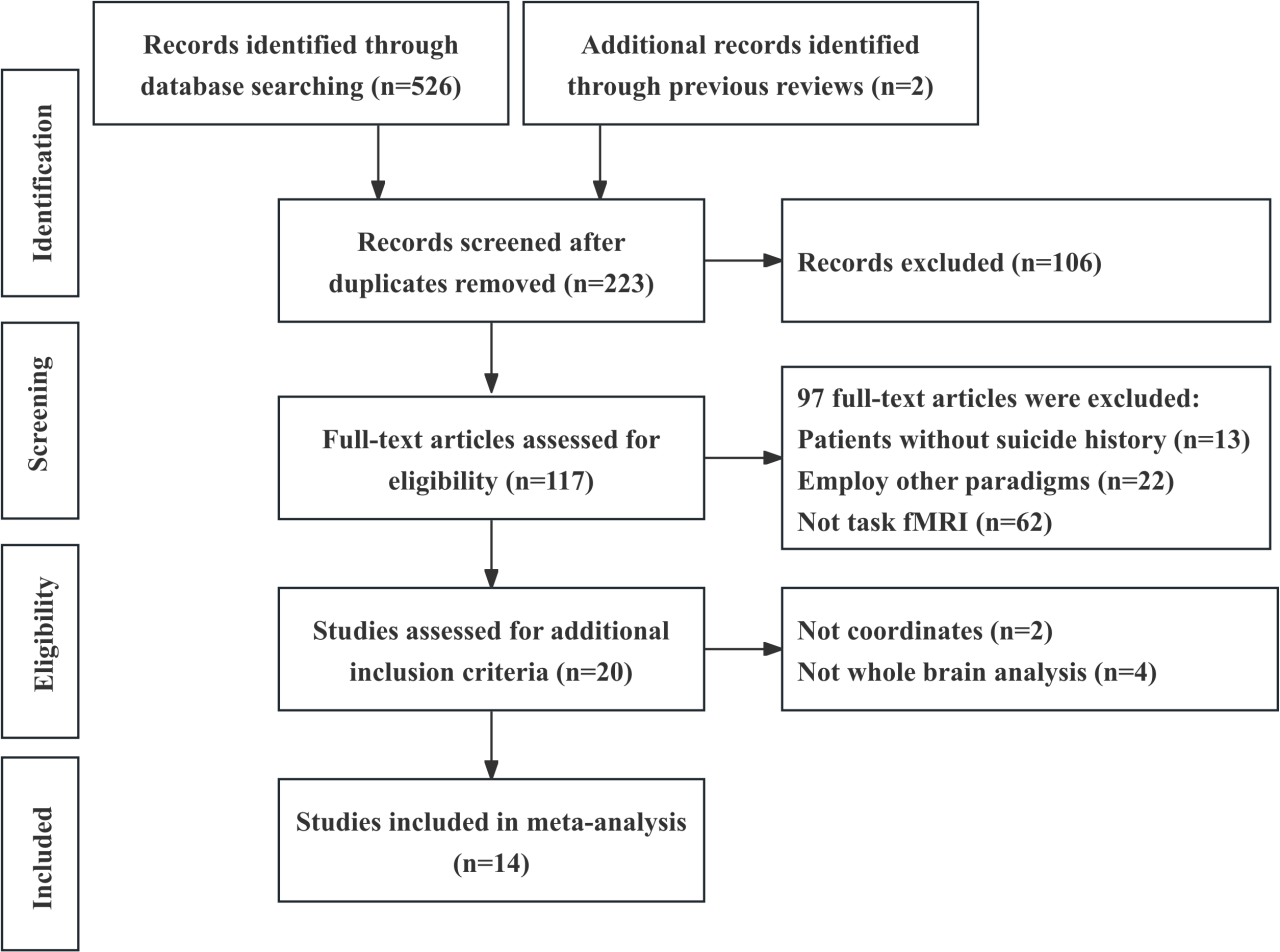
Preferred Reporting Items for Systematic Reviews and Meta-Analyses (PRISMA) flowchart.

A total of 14 studies (25–38), comprising 293 patients with STB and 414 PCs, were included in the main meta-analysis (**Figure 1**). It is worth mentioning that three studies were removed from the list of possibly included studies. Two lacked reported brain coordinates (39, 40), and one (41) involved patients who were immediate biological relatives of individuals who had died by suicide. There were no significant differences in the age of the patients with STB (29.36 ± 15.37 years) and age of the PCs (29.92 ± 16.18 years) (t = -0.10, p = 0.93) nor in their sex (female proportion of 52.21% for patients with STB and 51.93% for PCs, χ^2^ = 0.006, p = 0.94). In total, there are 8 fMRI studies based on DM tasks (performed by 183 patients with STB and 229 PCs) and 6 fMRI studies based on EF tasks (performed by 110 patients with STB and 185 PCs). Thirteen studies were performed using a 3.0 T MRI system and one study used a 1.5 T MRI system. The voxel size of the acquired images ranged from an isotropic voxel size of 2.5 mm to 3.75 mm. Clinical characteristics and demographic information for the participants are presented in **Table 1**, and task type, analysis software, magnetic field strength, voxel size, threshold, and quality score for each study are presented in **Supplementary Table S3** of the **Supplementary Materials**. Information regarding medication that was being taken by the patients is presented in **Supplementary Table S4** of the **Supplementary Materials**. The mean quality score was 8.5 out of 10 items (range, 7-9.5) (**Supplementary Table S3**).

**Table 1:** Details of the demographic and clinical characteristics of the studies included in the meta-analysis.

Study	Suicidal patients					Controls			Tasks#
	Type of patients	Suicide scale	Diagnosis	No. (female)	Mean age (years)	Type of controls	No. (female)	Mean age (years)	
Ai et al. (25)	SA	SSI, QSA	MDD+ANX	18 (14)	37.72	SI+PC	85 (50)	37.09	Tower of London task (EF)
Baek et al. (26)	SA	BHS, BSI	MDD	45 (19)	24.50	PC	47 (25)	26.80	Risk aversion task (DM)
Bomyea et al. (27)	SI	BDI-II	PTSD	9 (0)	30.89	PC	14 (0)	36.79	Working memory task (EF)
Dir et al. (28)	SI	^#^Suicidality items	ADHD+DBDs	22 (10)	11.88	PC	35 (11)	11.91	Balloon analogue risk task (DM)
Gorka et al. (37)	SI	C-SSRS	MDD*	39 (28)	18.6	PC*	69 (43)	18.4	Reward anticipation task (DM)
Gifuni et al. (38)	SA	C-SSRS	MDD	29(25)	16.3	PC	35(28)	16.0	Go-Nogo task (EF)
Ji et al. (29)	SA	BSI	MDD	23 (15)	21.39	PC	30 (18)	23.36	Balloon analogue risk task (DM)
Jollant et al. (30)	SA	RRRS, SIS	MDD	13 (0)	38.00	PC	12 (0)	43.00	Iowa gambling task (DM)
Matthews et al. (31)	SI	CSR	PTSD+MDD	13 (0)	29.54	PC	13 (0)	27.08	Stop signal task (EF)
Pan et al. (33)	SA	SIS, CSH, CSH, LRS,	MDD	15 (11)	16.20	PC	15 (8)	15.87	Go-Nogo task (EF)
Pan et al. (32)	SA	SIS	MDD	15 (11)	16.20	PC	14 (7)	15.79	Iowa gambling task (DM)
Potvin et al. (34)	SA	C-SSRS	Schizophrenia	13 (0)	39.10	PC	9 (0)	32.10	Balloon analogue risk task (DM)
Richard-devantoy et al. (36)	SA	SIS	MDD	26 (15)	40.30	PC	23 (15)	41.30	Go-Nogo task (EF)
Vanyukov et al. (35)	SA	BSI	MDD	13 (5)	70.38	PC	13 (10)	73.38	Delay discounting task (DM)

# Suicidality items assessed lifetime history of the following: 1) ideation; 2) intent; or 3) nonsuicidal self-harm.

* The SI group (7.7%) and the control group (2.9%) were matched on current MDD diagnosis.

#In this meta-analysis, EF covers working memory, inhibition, and DM covers risk decision-making, time preference decision-making, and the reward anticipation task.

SI, psychiatric patients with suicide ideation; SA, psychiatric patients with suicide attempts; PC, psychiatric patients without suicide ideation or attempts; BDI-II, Beck Depression Inventory-II; BHS, the Beck Hopelessness Scale; BSI, Beck Scale for Suicide Ideation; CSH, Columbia Suicide History Form; CSR, Comprehensive Suicide Risk Assessment; C-SSRS, Columbia Suicide Severity Rating Scale; FIGS, The Family Interview for Genetics Studies; LRS, Lethality Rating Scale; RRRS, Risk Rescue Rating Scale; SIS, the Suicide Intent Scale; SIQ, Suicidal Ideation Questionnaire; SSI, Scale for Suicide Ideation, QSA, Question on Suicide Attempts; MDD, major depressive disorders; ANX, anxiety disorder; PTSD, post-traumatic stress disorder; ADHD, attention deficit and hyperactivity disorder; DBD, disruptive behavior disorder; EF, executive function.

### 3.2 Meta-analysis of DM tasks

**Figure 2:**
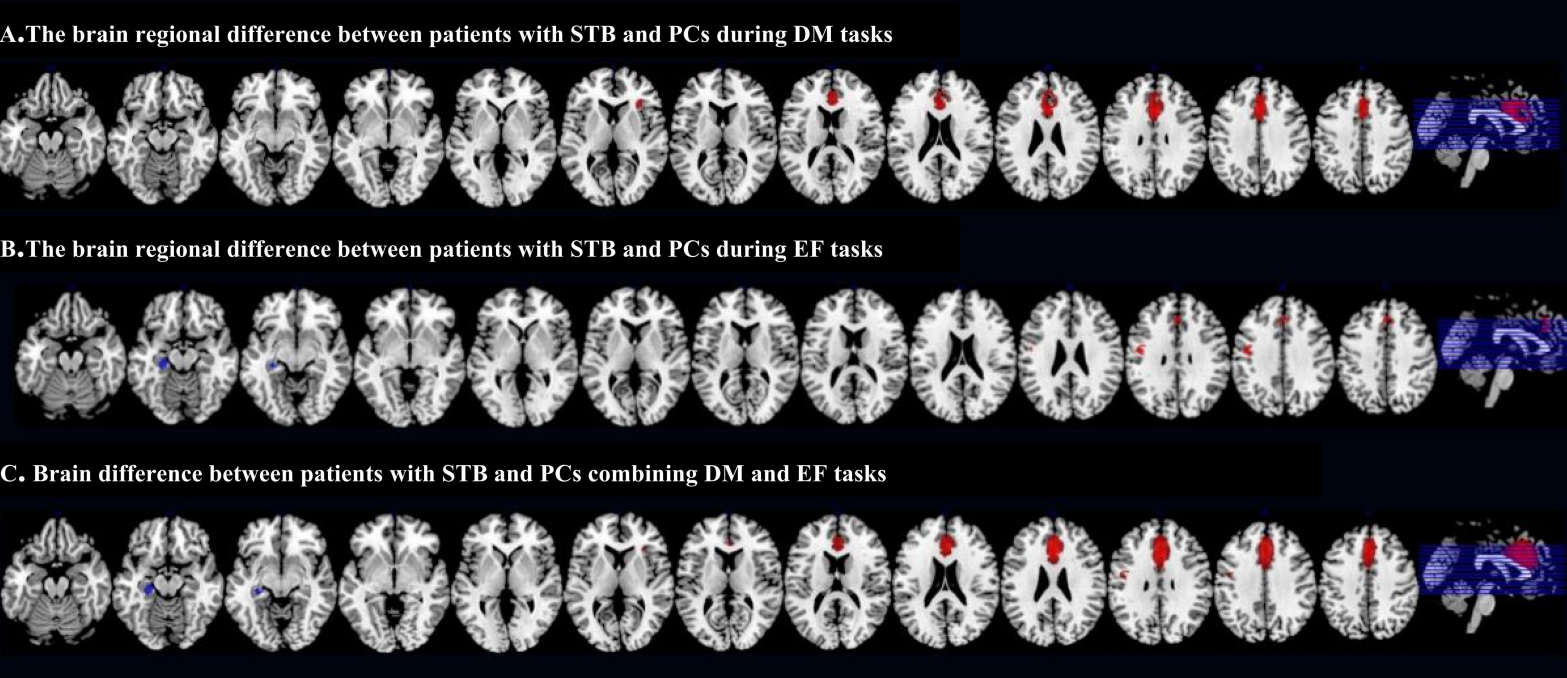
Between-group analysis of brain region alteration in patients with STB compared with PCs **(A)**. During DM tasks; **(B)**. During EF tasks; **(C)**. Multimodal analysis combining DM and EF tasks. Red: activation; Blue: deactivation; L, left; R, right; STB, suicide thoughts and behaviors; PCs, Patient controls; DM, Decision making; EF, Executive function.

During the DM processing, patients with STB showed hyper-activation in left ACC (BA24, p = 0.000371) and right insula (p = 0.000640), and hypo-activation in left insula (BA 48, p = 0.000387) and left hippocampus (Brodmann area 20, p = 0.0000619) compared to PCs (**Table 2** and **Figure 2**).

**Table 2:** Regional differences in activations between patients with STB and patient controls during DM or EF tasks.

Brain region	MNI coordinates of cluster peak (mm)			Number of voxels	SDM-Z score	*p value*
	x	y	z			
**Decision-making (n = 8)**						
*Patients with STB > patient controls*						
L anterior cingulate cortex, BA 24	-2	28	24	876	1.469	*0.000371*
R insula, BA 48	30	28	6	40	1.443	*0.000640*
*Patients with STB < patient controls*						
L insula, BA 48	-34	-20	-4	960	-1.271	*0.000387*
L hippocampus, BA 20	-30	-24	-8	201	-1.468	*0.0000619*
**Executive function (n = 6)**						
*Patients with STB > patient controls*						
L anterior cingulate cortex, BA24	-2	16	28	562	1.509	*0.00121*
L precentral gyrus, BA4	-56	-8	28	41	1.268	*0.00391*
*Patients with STB < patient controls*						
None						
**Suicide Attempt (n = 9)**						
*Patients with STB > patient controls*						
None						
*Patients with STB < patient controls*						
L hippocampus, BA 20	-30	-24	-8	193	-1.497	0.000031
**Suicide ideation (n = 4)**						
*Patients with STB > patient controls*						
L anterior cingulate cortex, BA 24	2	18	34	1225	2.163	0.000052
R insula, BA 48	32	28	8	79	2.074	0.000134
Patients with STB < patient controls						
None						

MNI, Montreal Neurological Institute; SDM, Seed-based d Mapping; STB, Suicide Thoughts and Behaviors; R, Right; L, Left; pos: positive; neg: negative.

### 3.3 Meta-analysis of EF tasks

During the EF processing, patients with STB showed hyper-activation in left ACC (BA24, p = 0.00121) and left precentral gyrus (p = 0.00391) compared to PCs, and no hypo-activated brain regions were found (**Table 2** and **Figure 2**).

### 3.4 Uniformity analysis of DM and EF tasks

To evaluate the consistent activated abnormal brain regions of the DM and EF tasks, we made a multimodal analysis, and we found that right ACC was increased in DM and EF with good agreement, while left insula was decreased in DM and increased in EF, and no brain regions with decreased activation in DM and EF were found. For enhanced clarity, we have condensed all main outcomes and uniformity analysis findings into **Table 3**.

**Table 3:** Summary of analysis results.

	Left anterior cingulate cortex	Right insula	Left precentral	Left insula	Left hippocampus
DM tasks	↑	↑	–	↓	↓
EF tasks	↑	–	↑	–	–
DM_pos_EF_pos_	✓	–	–	–	–
DM_pos_EF_neg_	–	–	–	–	–
DM_neg_EF_pos_	–	–	–	✓	–
DM_neg_EF_neg_	–	–	–	–	–

DM, decision making; EF, executive function; pos, positive; neg, negative.

### 3.5 Sensitivity analysis and publication bias

Results of the whole-brain jack-knife sensitivity analysis are presented in **Supplementary Table S5** of the **Supplementary Materials**. The brain regions from meta-analysis of DM tasks were more stable than those of EF tasks across study combination. Evidence of publication bias was not found for any of the brain regions for which effects are reported (**Supplementary Table S6**, p > 0.05).

### 3.6 Meta-regression and subgroup analyses

**Figure 3:**
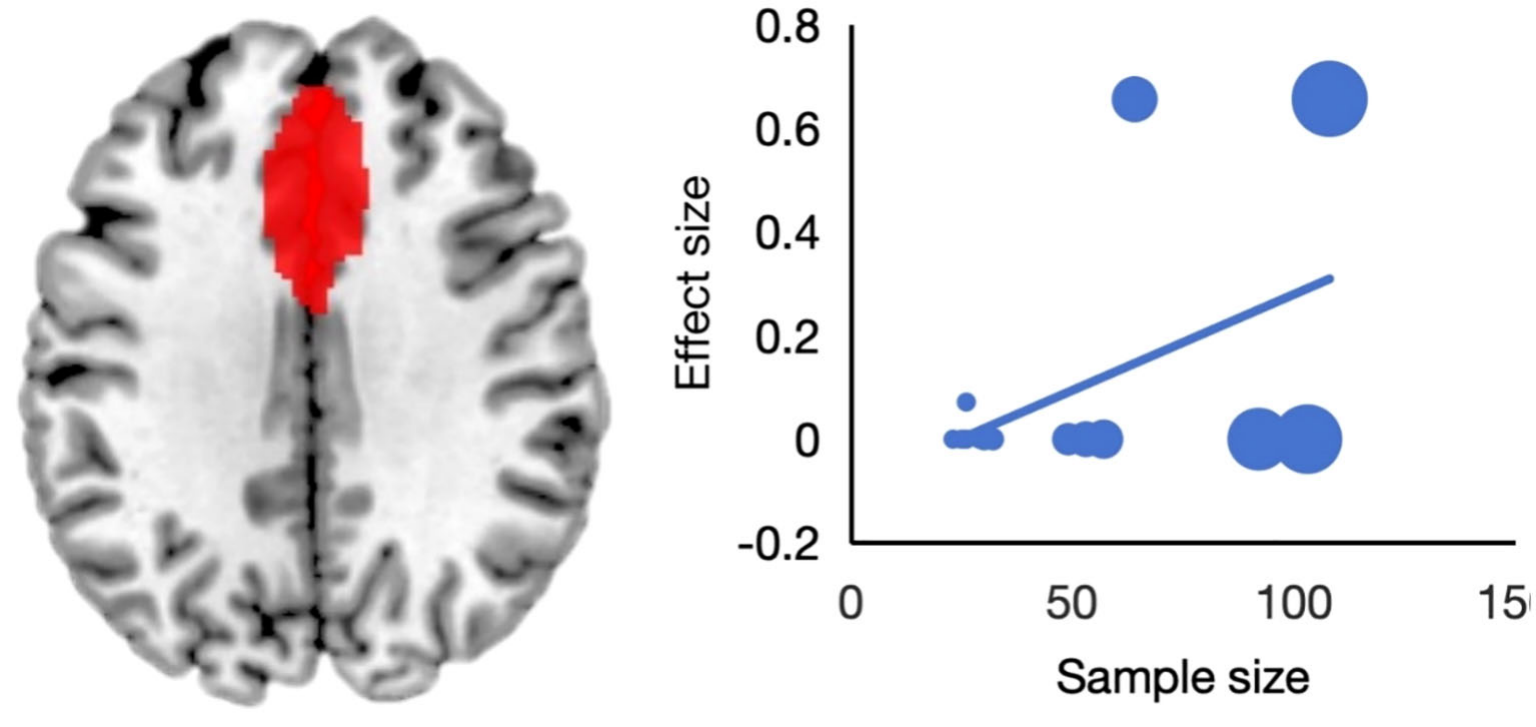
The meta-regression analysis revealing a significant association between sample size and brain activation in the right anterior cingulate cortex.

Through meta-regression analysis, we did not find any significant effects of mean age and the percentage of female patients in the study cohort. However, the size of the cohort of patients with STB was associated with gray matter activation in left ACC (MNI coordinates: x = 4, y = 30, z = 26, SDM = 1.7, p = ~0) (**Figure 3**). To assess the impact of sample size, we divided the studies into two groups: those with over 15 participants showed significant ACC activation, while those with 15 or fewer did not. The subgroup analysis suggested that the activation pattern in SI more closely resembles that of the DM task results, though the overall extent of brain impairment appears less pronounced than in the SA subgroup (**Table 2**).

## 4 Discussion

Understanding the neuropsychological mechanisms underlying DM and EF processes in patients with STB is crucial for effective risk assessment and intervention strategies. Establishing a cohesive framework that integrates DM and EF is crucial for interpreting the study’s findings. Moreover, a detailed examination of distinct regional contributions remains essential for a comprehensive understanding of the underlying neural mechanisms. For example, the ACC is a commonly activated area implicated in both cognitive functions. Although the left insula does not exhibit significant activation in EF, our multimodal analysis reveals that it is an abnormal brain region shared by the two tasks.

### 4.1 Altered neural circuitry across DM and EF tasks

In this study, we identified altered neural activity in STB patients in ACC was implicated in both DM and EF, suggesting a shared neurocognitive disruption. The left ACC and right insula exhibited hyperactivation in DM task, which forms key nodes of the Salience Network (42). During DM, hyperactivation in these regions suggests heightened sensitivity to negatively valenced information and impaired assessment of positive outcomes, potentially leading to altered risk perception and preferential engagement in avoidance behaviors (43). Conversely, hypoactivation in the Default Mode Network, i.e., left insula and hippocampus may reflect disorganized affective integration and contextual memory retrieval, further compromising adaptive DM (44).

Concurrently, we observed increased activation in the ACC and precentral gyrus during EF tasks, indicating enhanced effort toward impulse control in patients with STB, compared with patient controls (42). This common ACC engagement across both domains’ points to its central role in monitoring conflict and regulating emotional and cognitive responses.

The hyperactivation in Salience Network and suppression of Default Mode Network activity suggest a persistently alert, control-oriented cognitive state—even long after the STB episode—which may reflect enduring difficulties in disengaging from threat-related processing and adjusting behavioral responses, supporting these stable aberrations in regions is a potential neurobiological trait marker of STB.

### 4.2 Role of anterior cingulate gyrus in DM and EF tasks

Our study found that the left ACC was hyperactivated while STB patients performing the DM and EF tasks. The ACC plays a pivotal role in EF and DM processes, particularly during complex cognitive tasks and emotional feedback processing (45, 46). Neuroimaging studies have revealed distinct activation patterns in the ACC across varying psychological states and task conditions, providing critical insights into the cognitive-emotional mechanisms of STB. For example, research demonstrates that suicide attempters exhibit functional abnormalities in the prefrontal cortex and cingulate gyrus during DM tasks, potentially impairing their capacity for reward-based DM under emotional contexts (47). These dysfunctions may correlate with hyperactivation of the ACC during emotional information processing and executive control (48). Moreover, the ACC shows altered functional connectivity with other key brain regions during these processes. For instance, ACC-orbitofrontal cortex synchronization plays an essential role in value-guided DM, with enhanced coupling observed during high-reward selections (49). Such connectivity disturbances may underlie the DM impairments characteristic of suicide attempters (50). In summary, the observed ACC hyperactivity during EF and DM tasks likely reflects core cognitive-emotional dysregulation in STB (51). Further investigation of state-dependent ACC functional dynamics could advance our understanding of suicidal neuropathology and inform novel clinical interventions.

In prior research abnormalities in mPFC have been reported in patients with psychiatric disorders associated with suicide risk, such as major depressive disorder and bipolar disorder (52). In addition, Prior research undertaken by our team (53) in which increased activation of mPFC was reported in patients with alcohol use disorder during response inhibition tasks, aligns with the findings of the present study. However, this study did not find the impaired activation in the above brain regions. Further exploration is needed to determine whether these regions are critically implicated in suicide risk through whole-brain studies.

### 4.3 Impairment of hippocampus during DM tasks

The significant hypo-activation of left hippocampus in patients with STB compared to PCs when performing DM tasks, was not observed for EF task, suggesting that there may be a distinct neural signature associated with DM processes in individuals with a history of STB (54). The hippocampus plays a crucial role in guiding the selection of appropriate DM strategies (55–57). This observation is in line with a prior review highlighting the importance of enhancing cognitive functions involving the hippocampus for effective suicide prevention (58). Beyond its functional role, the hippocampus has been implicated in psychopathological changes associated with suicide, whereby the heightened sensitivity of the hippocampus to stress represents a notable risk factor contributing to STB (59).

The fact that hypo-activation of the hippocampus was not observed during EF tasks points to a task-specific deficit rather than a global reduction in neural activity and suggests that cognitive impairments in patients with STB may be domain-specific (60). This finding is consistent with prior research showing that cognitive impairments in suicide attempters tend to be more pronounced in tasks requiring higher-order cognitive abilities (8).

### 4.4 Roles of insula in DM tasks

The meta-analysis has revealed there to be significantly dysregulated in insula activation patterns between patients with STB and PCs during performance of DM tasks. The bilateral insula has been reported to play a crucial role in processing of negative emotions, interoceptive awareness and risk assessment (61). Increased activation in the right insula during DM tasks has been suggested to reflect heightened emotional reactivity and difficulty in processing negative emotional stimuli, potentially contributing to impulsivity and risky DM (62). The left insula is closely related to positive emotions (63), and impaired activation in this brain area during the DM task in patients with STB suggests that the response to potential rewards is weakened, affecting the motivation of DM (64).

In addition, our multimodal analysis also found that in DM, left insula also showed a new result, namely that this brain region also increased activation in EF. As a new result in the multimodal analysis, we need to be cautious about this result, although studies show that the left insula can participate in the dynamic regulation of executive functional networks through functional connectivity with other brain regions (e. g., prefrontal cortex, anterior cingulate, and parietal cortex) (65).

### 4.5 Role of precentral cortex in EF tasks

The current study identified increased activation in the precentral gyrus during EF tasks, aligning with the dual role of the precentral cortex in motor planning and higher cognitive functions (66). While it is a critical component of the primary motor cortex, the precentral cortex also engages in cognitive control, response inhibition, and attentional regulation through its functional connectivity with the prefrontal cortex (67). Neuroimaging studies indicate that the precentral cortex collaborates with the prefrontal cortex during EF tasks to convert cognitive control signals into motor outputs, particularly in scenarios requiring rapid response inhibition and task switching (68). Additionally, as a pivotal node in the mirror neuron system (69), the precentral cortex may further support its involvement in higher cognitive functions, such as theory of mind and empathy. Impairment of this region may result in deficits in EF, particularly in motor planning and cognitive control tasks (70). These findings underscore the significance of the precentral cortex within EF networks, offering new insights into its role in prefrontal-motor cortical circuits (71).

### 4.6 Limitations

While providing novel insights, this meta-analysis has several important limitations that warrant cautious interpretation of the findings: First, our reliance on reported peak coordinates and effect sizes (rather than raw statistical maps) inherently reduces spatial precision and may obscure subtle but clinically relevant neural patterns. This methodological approach, while common in neuroimaging meta-analyses, prevents more nuanced characterization of neural circuitry. Second, the inclusion of patients with varied comorbid psychiatric disorders introduces important clinical heterogeneity. This diversity in psychiatric comorbidities may have potentially obscured suicide-related neural features that are independent of primary diagnoses. These inherent clinical variations suggest our findings likely reflect shared transdiagnostic vulnerabilities to suicidal behavior across psychiatric disorders rather than suicide-specific neural markers. Third, the cross-sectional nature of included studies fundamentally limits causal inference regarding whether the observed neural alterations represent predisposing vulnerabilities or consequences of suicidal states. Longitudinal designs with repeated assessments will be crucial for disentangling these temporal relationships.

## 5 Conclusions

This meta-analysis reveals distinct neural patterns in STB patients, with DM-related regions showing hypoactivation—indicating impaired reward and risk processing—and DM/EF-related regions exhibiting hyperactivation, which may reflect compensatory efforts or increased cognitive demand. As a common activated region in both DM and EF tasks, the cingulate gyrus emerges as a key hub linking these domains, underscoring its role in integrating these two cognitive processes. These dysfunctions may underlie the core pathophysiology of DM and EF deficits in patients with a history of STB.

## Data Availability

The raw data supporting the conclusions of this article will be made available by the authors, without undue reservation.

## References

[1] Organization GWH . Suicide worldwide in 2019: global health estimates. Geneva: World Health Organization (2021), 35. https://www.who.int/publications/i/item/9789240026643.

[2] Riera-Serra P Gili M Navarra-Ventura G Riera-López Del Amo A Montaño JJ Coronado-Simsic V . Longitudinal associations between executive function impairments and suicide risk in patients with major depressive disorder: A 1-year follow-up study. Psychiatry Res. (2023) 325:115235. doi: 10.1016/j.psychres.2023.115235, PMID: 37178501

[3] Fernandez-Sevillano J Alberich S Zorrilla I Gonzalez-Ortega I Lopez MP Perez V . Cognition in recent suicide attempts: altered executive function. Front Psychiatry. (2021) 12:12. doi: 10.3389/fpsyt.2021.701140, PMID: 34366931 PMC8339467

[4] Wang H Zhu R Dai Z Tian S Shao J Wang X . Aberrant functional connectivity and graph properties in bipolar II disorder with suicide attempts. J Affect Disord. (2020) 275:202–9. doi: 10.1016/j.jad.2020.07.016, PMID: 32734909

[5] Allen KJD Bozzay ML Edenbaum ER . Neurocognition and suicide risk in adults. Curr Behav Neurosci Rep. (2019) 6:151–65. doi: 10.1007/s40473-019-00189-y

[6] Ullsperger M Danielmeier C . Motivational and Cognitive Control: From motor inhibition to social decision making. Neurosci Biobehav Rev. (2022) 136:104600. doi: 10.1016/j.neubiorev.2022.104600, PMID: 35248675

[7] Sastre-Buades A Alacreu-Crespo A Courtet P Baca-Garcia E Barrigon ML . Decision-making in suicidal behavior: A systematic review and meta-analysis. Neurosci Biobehav Rev. (2021) 131:642–62. doi: 10.1016/j.neubiorev.2021.10.005, PMID: 34619171

[8] Escobar LE Liew M Yirdong F Mandelos KP Ferraro-Diglio SR Abraham BM . Reduced attentional control in individuals with a history of suicide attempts compared to those with suicidal ideation: Results from a systematic review and meta-analysis. J Affect Disord. (2024) 349:8–20. doi: 10.1016/j.jad.2023.12.082, PMID: 38169241

[9] Gifuni AJ Perret LC Lacourse E Geoffroy MC Mbekou V Jollant F . Decision-making and cognitive control in adolescent suicidal behaviors: a qualitative systematic review of the literature. Eur Child Adolesc Psychiatry. (2021) 30:1839–55. doi: 10.1007/s00787-020-01550-3, PMID: 32388626

[10] Perrain R Dardennes R Jollant F . Risky decision-making in suicide attempters, and the choice of a violent suicidal means: an updated meta-analysis. J Affect Disord. (2021) 280:241–9. doi: 10.1016/j.jad.2020.11.052, PMID: 33220560

[11] Brown VM Wilson J Hallquist MN Szanto K Dombrovski AY . Ventromedial prefrontal value signals and functional connectivity during decision-making in suicidal behavior and impulsivity. Neuropsychopharmacol. (2020) 45:1034–41. doi: 10.1038/s41386-020-0632-0, PMID: 32035425 PMC7162923

[12] Olié E Ding Y Le Bars E de Champfleur NM Mura T Bonafé A . Processing of decision-making and social threat in patients with history of suicidal attempt: A neuroimaging replication study. Psychiatry Res: Neuroimaging. (2015) 234:369–77. doi: 10.1016/j.pscychresns.2015.09.020, PMID: 26483212

[13] Monkul ES Hatch JP Nicoletti MA Spence S Brambilla P Lacerda AL . Fronto-limbic brain structures in suicidal and non-suicidal female patients with major depressive disorder. Mol Psychiatry. (2007) 12:360–6. doi: 10.1038/sj.mp.4001919, PMID: 17389903

[14] Minzenberg MJ Lesh TA Niendam TA Yoon JH Cheng Y Rhoades RN . Control-related frontal-striatal function is associated with past suicidal ideation and behavior in patients with recent-onset psychotic major mood disorders. J Affect Disord. (2015) 188:202–9. doi: 10.1016/j.jad.2015.08.049, PMID: 26363618

[15] Malhi GS Das P Outhred T Bryant RA Calhoun V Mann JJ . Default mode dysfunction underpins suicidal activity in mood disorders. Psychol Med. (2020) 50:1214–23. doi: 10.1017/S0033291719001132, PMID: 31144614

[16] Huber RS McGlade EC Legarreta M Subramaniam P Renshaw PF Yurgelun-Todd DA . Cingulate white matter volume and associated cognitive and behavioral impulsivity in Veterans with a history of suicide behavior. J Affect Disord. (2021) 281:117–24. doi: 10.1016/j.jad.2020.11.126, PMID: 33316716

[17] Feredoes E Postle BR . Localization of load sensitivity of working memory storage: quantitatively and qualitatively discrepant results yielded by single-subject and group-averaged approaches to fMRI group analysis. Neuroimage. (2007) 35:881–903. doi: 10.1016/j.neuroimage.2006.12.029, PMID: 17296315 PMC1994574

[18] Chen CF Chen WN Zhang B . Functional alterations of the suicidal brain: a coordinate-based meta-analysis of functional imaging studies. Brain Imaging Behav. (2022) 16:291–304. doi: 10.1007/s11682-021-00503-x, PMID: 34351557

[19] Li H Chen Z Gong Q Jia Z . Voxel-wise meta-analysis of task-related brain activation abnormalities in major depressive disorder with suicide behavior. Brain Imaging Behav. (2020) 14:1298–308. doi: 10.1007/s11682-019-00045-3, PMID: 30790165

[20] Auerbach RP Pagliaccio D Allison GO Alqueza KL Alonso MF . Neural correlates associated with suicide and nonsuicidal self-injury in youth. Biol Psychiatry. (2021) 89:119–33. doi: 10.1016/j.biopsych.2020.06.002, PMID: 32782140 PMC7726029

[21] Lancaster JL Tordesillas-Gutiérrez D Martinez M Salinas F Evans A Zilles K . Bias between MNI and Talairach coordinates analyzed using the ICBM-152 brain template. Hum Brain Mapp. (2007) 28:1194–205. doi: 10.1002/hbm.20345, PMID: 17266101 PMC6871323

[22] Radua J Mataix-Cols D Phillips ML El-Hage W Kronhaus DM Cardoner N . A new meta-analytic method for neuroimaging studies that combines reported peak coordinates and statistical parametric maps. Eur Psychiatry. (2012) 27:605–11. doi: 10.1016/j.eurpsy.2011.04.001, PMID: 21658917

[23] Radua J Rubia K Canales-Rodríguez EJ Pomarol-Clotet E Fusar-Poli P Mataix-Cols D . Anisotropic kernels for coordinate-based meta-analyses of neuroimaging studies. Front Psychiatry. (2014) 5:13. doi: 10.3389/fpsyt.2014.00013, PMID: 24575054 PMC3919071

[24] Egger M Davey Smith G Schneider M Minder C . Bias in meta-analysis detected by a simple, graphical test. BMJ. (1997) 315:629–34. doi: 10.1136/bmj.315.7109.629, PMID: 9310563 PMC2127453

[25] Ai H van Tol MJ Marsman JC Veltman DJ Ruhé HG van der Wee NJA . Differential relations of suicidality in depression to brain activation during emotional and executive processing. J Psychiatr Res. (2018) 105:78–85. doi: 10.1016/j.jpsychires.2018.08.018, PMID: 30212727

[26] Baek K Kwon J Chae JH Chung YA Kralik JD Min JA . Heightened aversion to risk and loss in depressed patients with a suicide attempt history. Sci Rep. (2017) 7:11228. doi: 10.1038/s41598-017-10541-5, PMID: 28894106 PMC5593974

[27] Bomyea J Stout DM Simmons AN . Attenuated prefrontal and temporal neural activity during working memory as a potential biomarker of suicidal ideation in veterans with PTSD. J Affect Disord. (2019) 257:607–14. doi: 10.1016/j.jad.2019.07.050, PMID: 31349177

[28] Dir AL Allebach CL Hummer TA Adams ZW Aalsma MC Finn PR . Atypical cortical activation during risky decision making in disruptive behavior disordered youths with histories of suicidal ideation. Biol Psychiatry Cognit Neurosci Neuroimaging. (2020) 5:510–9. doi: 10.1016/j.bpsc.2019.10.016, PMID: 32007432 PMC10568982

[29] Ji X Zhao J Li H Pizzagalli DA Law S Lin P . From motivation, decision-making to action: An fMRI study on suicidal behavior in patients with major depressive disorder. J Psychiatr Res. (2021) 139:14–24. doi: 10.1016/j.jpsychires.2021.05.007, PMID: 34004553

[30] Jollant F Lawrence NS Olie E O’Daly O Malafosse A Courtet P . Decreased activation of lateral orbitofrontal cortex during risky choices under uncertainty is associated with disadvantageous decision-making and suicidal behavior. NeuroImage. (2010) 51:1275–81. doi: 10.1016/j.neuroimage.2010.03.027, PMID: 20302946

[31] Matthews S Spadoni A Knox K Strigo I Simmons A . Combat-exposed war veterans at risk for suicide show hyperactivation of prefrontal cortex and anterior cingulate during error processing. Psychosom Med. (2012) 74:471–5. doi: 10.1097/PSY.0b013e31824f888f, PMID: 22511726 PMC4018224

[32] Pan L Segreti A Almeida J Jollant F Lawrence N Brent D . Preserved hippocampal function during learning in the context of risk in adolescent suicide attempt. Psychiatry Res. (2013) 211:112–8. doi: 10.1016/j.pscychresns.2012.07.008, PMID: 23158778 PMC3570719

[33] Pan LA Batezati-Alves SC Almeida JRC Segreti A Akkal D Hassel S . Dissociable patterns of neural activity during response inhibition in depressed adolescents with and without suicidal behavior. J Am Acad Child Adolesc Psychiatry. (2011) 50:602–611.e603. doi: 10.1016/j.jaac.2011.03.018, PMID: 21621144 PMC3104246

[34] Potvin S Tikàsz A Richard-Devantoy S Lungu O Dumais A . History of Suicide Attempt Is Associated with Reduced Medial Prefrontal Cortex Activity during Emotional Decision-Making among Men with Schizophrenia: An Exploratory fMRI Study. Schizophr Res Treat. (2018) 2018:1–8. doi: 10.1155/2018/9898654, PMID: 29686902 PMC5852894

[35] Vanyukov PM Szanto K Hallquist MN Siegle GJ Reynolds CF Forman SD . Paralimbic and lateral prefrontal encoding of reward value during intertemporal choice in attempted suicide. Psychol Med. (2015) 46:381–91. doi: 10.1017/S0033291715001890, PMID: 26446615 PMC4797639

[36] Richard-Devantoy S Szanto K Butters MA Kalkus J Dombrovski AY . Cognitive inhibition in older high-lethality suicide attempters. Int J Geriatr Psychiatry. (2015) 30:274–83. doi: 10.1002/gps.4138, PMID: 24816626 PMC4229451

[37] Gorka SM Manzler CA Jones EE Smith RJ Bryan CJ . Reward-related neural dysfunction in youth with a history of suicidal ideation: The importance of temporal predictability. J Psychiatr Res. (2023) 158:20–6. doi: 10.1016/j.jpsychires.2022.11.036, PMID: 36549196

[38] Gifuni AJ Pereira F Chakravarty MM Lepage M Chase HW Geoffroy MC . Perception of social inclusion/exclusion and response inhibition in adolescents with past suicide attempt: a multidomain task-based fMRI study. Mol Psychiatry. (2024) 29:2135–44. doi: 10.1038/s41380-024-02485-w, PMID: 38424142

[39] Siegel A Zhang H Wei X Tao H Mwansisya TE Pu W . Opposite effective connectivity in the posterior cingulate and medial prefrontal cortex between first-episode schizophrenic patients with suicide risk and healthy controls. PloS One. (2013) 8:e63477. doi: 10.1371/journal.pone.0063477, PMID: 23704911 PMC3660523

[40] Dombrovski AY Szanto K Clark L Reynolds CF Siegle GJ . Reward signals, attempted suicide, and impulsivity in late-life depression. JAMA Psychiatry. (2013) 70:1020. doi: 10.1001/jamapsychiatry.2013.75, PMID: 23925710 PMC3859132

[41] Ding Y Pereira F Hoehne A Beaulieu MM Lepage M Turecki G . Altered brain processing of decision-making in healthy first-degree biological relatives of suicide completers. Mol Psychiatry. (2016) 22:1149–54. doi: 10.1038/mp.2016.221, PMID: 27956745

[42] Sridharan D Levitin DJ Menon V . A critical role for the right fronto-insular cortex in switching between central-executive and default-mode networks. Proc Natl Acad Sci U S A. (2008) 105:12569–74. doi: 10.1073/pnas.0800005105, PMID: 18723676 PMC2527952

[43] Uddin LQ . Salience processing and insular cortical function and dysfunction. Nat Rev Neurosci. (2015) 16:55–61. doi: 10.1038/nrn3857, PMID: 25406711

[44] Yan CG Chen X Li L Castellanos FX Bai TJ Bo QJ . Reduced default mode network functional connectivity in patients with recurrent major depressive disorder. Proc Natl Acad Sci U S A. (2019) 116:9078–83. doi: 10.1073/pnas.1900390116, PMID: 30979801 PMC6500168

[45] Bush G Luu P Posner MI . Cognitive and emotional influences in anterior cingulate cortex. Trends Cognit Sci. (2000) 4:215–22. doi: 10.1016/S1364-6613(00)01483-2, PMID: 10827444

[46] Elliott R Sahakian BJ McKay AP Herrod JJ Robbins TW Paykel ES . Neuropsychological impairments in unipolar depression: the influence of perceived failure on subsequent performance. Psychol Med. (1996) 26:975–89. doi: 10.1017/S0033291700035303, PMID: 8878330

[47] Carter CS Botvinick MM Cohen JD . The contribution of the anterior cingulate cortex to executive processes in cognition. Rev Neurosci. (1999) 10:49–57. doi: 10.1515/REVNEURO.1999.10.1.49, PMID: 10356991

[48] Balewski ZZ Elston TW Knudsen EB Wallis JD . Value dynamics affect choice preparation during decision-making. Nat Neurosci. (2023) 26:1575–83. doi: 10.1038/s41593-023-01407-3, PMID: 37563295 PMC10576429

[49] Deng J Zhang M Chen G Lu X Cheng X Qin C . Exploring neural changes. Associated with suicidal ideation and attempts in major depressive disorder: A multimodal study. Brain Res Bull. (2025) 225:111336. doi: 10.1016/j.brainresbull.2025.111336, PMID: 40222622

[50] Alacreu-Crespo A Olié E Le Bars E Cyprien F Deverdun J Courtet P . Prefrontal activation in suicide attempters during decision making with emotional feedback. Transl Psychiatry. (2020) 10(1):313. doi: 10.1038/s41398-020-00995-z, PMID: 32948747 PMC7501865

[51] Fatahi Z Ghorbani A Ismail Zibaii M Haghparast A . Neural synchronization between the anterior cingulate and orbitofrontal cortices during effort-based decision making. Neurobiol Learn Mem. (2020) 175:107320. doi: 10.1016/j.nlm.2020.107320, PMID: 33010385

[52] Sheline YI . Neuroimaging studies of mood disorder effects on the brain. Biol Psychiatry. (2003) 54:338–52. doi: 10.1016/S0006-3223(03)00347-0, PMID: 12893109

[53] Cao Y Tian F Zeng J Gong Q Yang XandJia Z . The brain activity pattern in alcohol-use disorders under inhibition response Task. J Psychiatr Res. (2023) 163:127–34. doi: 10.1016/j.jpsychires.2023.05.009, PMID: 37209618

[54] Chase HW Auerbach RP Brent DA Posner J Weissman MM Talati A . Dissociating default mode network resting state markers of suicide from familial risk factors for depression. Neuropsychopharmacology. (2021) 46:1830–8. doi: 10.1038/s41386-021-01022-5, PMID: 34059799 PMC8358011

[55] Mızrak E Bouffard NR Libby LA Boorman ED Ranganath C . The hippocampus and orbitofrontal cortex jointly represent task structure during memory-guided decision making. Cell Rep. (2021) 37:110065. doi: 10.1016/j.celrep.2021.110065, PMID: 34852232 PMC8686644

[56] Bornstein AM Daw ND . Cortical and hippocampal correlates of deliberation during model-based decisions for rewards in humans. PLoS Comput Biol. (2013) 9:e1003387. doi: 10.1371/journal.pcbi.1003387, PMID: 24339770 PMC3854511

[57] Shohamy D Turk-Browne NB . Mechanisms for widespread hippocampal involvement in cognition. J Exp Psychol Gen. (2013) 142:1159–70. doi: 10.1037/a0034461, PMID: 24246058 PMC4065494

[58] Zhang L Lucassen PJ Salta E Verhaert PandSwaab DF . Hippocampal neuropathology in suicide: Gaps in our knowledge and opportunities for a breakthrough. Neurosci Biobehav Rev. (2022) 132:542–52. doi: 10.1016/j.neubiorev.2021.12.023, PMID: 34906612

[59] Vyas A Mitra R Shankaranarayana Rao BS Chattarji S . Chronic stress induces contrasting patterns of dendritic remodeling in hippocampal and amygdaloid neurons. J Neurosci. (2002) 22:6810–8. doi: 10.1523/JNEUROSCI.22-15-06810.2002, PMID: 12151561 PMC6758130

[60] da Silva RA Tancini MB Lage R Nascimento RL Santana CMT Landeira-Fernandez J . Autobiographical memory and episodic specificity across different affective states in bipolar disorder. Front Psychiatry. (2021) 12:641221. doi: 10.3389/fpsyt.2021.641221, PMID: 34025473 PMC8138163

[61] Meyer G . From the lateral edge to the center of the cortex: The development of the human insula. Neuroforum. (2018) 24:A151–8. doi: 10.1515/nf-2018-A008

[62] Craig AD . How do you feel? Interoception: the sense of the physiological condition of the body. Nat Rev Neurosci. (2002) 3:655–66. doi: 10.1038/nrn894, PMID: 12154366

[63] Craig AD . How do you feel–now? The anterior insula and human awareness. Nat Rev Neurosci. (2009) 10:59–70. doi: 10.1038/nrn2555, PMID: 19096369

[64] Pizzagalli DA Iosifescu D Hallett LA Ratner KG Fava M . Reduced hedonic capacity in major depressive disorder: evidence from a probabilistic reward task. J Psychiatr Res. (2008) 43:76–87. doi: 10.1016/j.jpsychires.2008.03.001, PMID: 18433774 PMC2637997

[65] Wang R Mo F Shen Y Song Y Cai H Zhu J . Functional connectivity gradients of the insula to different cerebral systems. Hum Brain Mapp. (2023) 44:790–800. doi: 10.1002/hbm.26099, PMID: 36206289 PMC9842882

[66] Dum RP Strick PL . Motor areas in the frontal lobe of the primate. Physiol Behav. (2002) 77:677–82. doi: 10.1016/S0031-9384(02)00929-0, PMID: 12527018

[67] Rae CL Hughes LE Anderson MC Rowe JB . The prefrontal cortex achieves inhibitory control by facilitating subcortical motor pathway connectivity. J Neurosci. (2015) 35:786–94. doi: 10.1523/JNEUROSCI.3093-13.2015, PMID: 25589771 PMC4293423

[68] Ridderinkhof KR Ullsperger M Crone EA Nieuwenhuis S . The role of the medial frontal cortex in cognitive control. Science. (2004) 306:443–7. doi: 10.1126/science.1100301, PMID: 15486290

[69] Rizzolatti G Craighero L . The mirror-neuron system. Annu Rev Neurosci. (2004) 27:169–92. doi: 10.1146/annurev.neuro.27.070203.144230, PMID: 15217330

[70] Gordon EM Chauvin RJ Van AN Rajesh A Nielsen A Newbold DJ . A somato-cognitive action network alternates with effector regions in motor cortex. Nature. (2023) 617:351–9. doi: 10.1038/s41586-023-05964-2, PMID: 37076628 PMC10172144

[71] Erika-Florence M Leech R Hampshire A . A functional network perspective on response inhibition and attentional control. Nat Commun. (2014) 5:4073. doi: 10.1038/ncomms5073, PMID: 24905116 PMC4059922

