# Supplementary material for: Neural correlates of decision making and executive function in suicidal thoughts and behaviors
Source: Front Psychiatry. 2025 Dec 10;16:1676986. doi: 10.3389/fpsyt.2025.1676986 (PMC12733375; doi:10.3389/fpsyt.2025.1676986)
Supplement: Supplementary file 1 [file Supplementaryfile1.pdf]

Supplementary materials

Table S1. PRISMA 2020 checklist

Section and Topic	Item #	Checklist item	Location where item is reported
**TITLE**			
Title	1	Identify the report as a systematic review.	Title page
**ABSTRACT**			
Abstract	2	See the PRISMA 2020 for Abstracts checklist.	**Abstract section**
**INTRODUCTION**			
Rationale	3	Describe the rationale for the review in the context of existing knowledge.	2nd para.of Introction
Objectives	4	Provide an explicit statement of the objective(s) or question(s) the review addresses.	Last para. of Introduction
METHODS			
Eligibility criteria	5	Specify the inclusion and exclusion criteria for the review and how studies were grouped for the syntheses.	Section 2.2 of Methods
Information sources	6	Specify all databases, registers, websites, organisations, reference lists and other sources searched or consulted to identify studies. Specify the date when each source was last searched or consulted.	Section 2.1 of Methods
Search strategy	7	Present the full search strategies for all databases, registers and websites, including any filters and limits used.	Section 2.1 of Methods
Selection process	8	Specify the methods used to decide whether a study met the inclusion criteria of the review, including how many reviewers screened each record and each report retrieved, whether they worked independently, and if applicable, details of automation tools used in the process.	Section 2.3 of Methods

Section and Topic	Item #	Checklist item	Location where item is reported
Data collection process	9	Specify the methods used to collect data from reports, including how many reviewers collected data from each report, whether they worked independently, any processes for obtaining or confirming data from study investigators, and if applicable, details of automation tools used in the process.	Section 2.3 of Methods
Data items	10a	List and define all outcomes for which data were sought. Specify whether all results that were compatible with each outcome domain in each study were sought (e.g. for all measures, time points, analyses), and if not, the methods used to decide which results to collect.	Section 2.4 of Methods
	10b	List and define all other variables for which data were sought (e.g. participant and intervention characteristics, funding sources). Describe any assumptions made about any missing or unclear information.	Table 1 and Table S3
Study risk of bias assessment	11	Specify the methods used to assess risk of bias in the included studies, including details of the tool(s) used, how many reviewers assessed each study and whether they worked independently, and if applicable, details of automation tools used in the process.	Section 2.5 of Methods
Effect measures	12	Specify for each outcome the effect measure(s) (e.g. risk ratio, mean difference) used in the synthesis or presentation of results.	Section 2.6 of Methods
Synthesis methods	13a	Describe the processes used to decide which studies were eligible for each synthesis (e.g. tabulating the study intervention characteristics and comparing against the planned groups for each synthesis (item #5)).	Section 2.5 of Methods
	13b	Describe any methods required to prepare the data for presentation or synthesis, such as handling of missing summary statistics, or data conversions.	Section 2.4 of Methods
	13c	Describe any methods used to tabulate or visually display results of individual studies and syntheses.	Section 2.5 of Methods
	13d	Describe any methods used to synthesize results and provide a rationale for the choice(s). If meta-analysis was performed, describe the model(s), method(s) to identify the presence and extent of statistical heterogeneity, and software package(s) used.	Section 2.5 of Methods

Section and Topic	Item #	Checklist item	Location where item is reported
	13e	Describe any methods used to explore possible causes of heterogeneity among study results (e.g. subgroup analysis, meta-regression).	Section 2.8 of Methods
	13f	Describe any sensitivity analyses conducted to assess robustness of the synthesized results.	Section 2.7 of Methods
Reporting bias assessment	14	Describe any methods used to assess risk of bias due to missing results in a synthesis (arising from reporting biases).	Section 2.7 of Methods
Certainty assessment	15	Describe any methods used to assess certainty (or confidence) in the body of evidence for an outcome.	Section 2.5 of Methods
**RESULTS**			
Study selection	16a	Describe the results of the search and selection process, from the number of records identified in the search to the number of studies included in the review, ideally using a flow diagram.	Section 3.1 of Results
	16b	Cite studies that might appear to meet the inclusion criteria, but which were excluded, and explain why they were excluded.	Section 3.1 of Results
Study characteristics	17	Cite each included study and present its characteristics.	Section 3.1 of Results
Risk of bias in studies	18	Present assessments of risk of bias for each included study.	Last sentence in section 3.2 of Results
Results of individual studies	19	For all outcomes, present, for each study: (a) summary statistics for each group (where appropriate) and (b) an effect estimate and its precision (e.g. confidence/credible interval), ideally using structured tables or plots.	Not appropriate
Results of syntheses	20a	For each synthesis, briefly summarise the characteristics and risk of bias among contributing studies.	Section 3.2 of Results
	20b	Present results of all statistical syntheses conducted. If meta-analysis was done, present for each the summary estimate	Table 2 and Table 3

Section and Topic	Item #	Checklist item	Location where item is reported
		and its precision (e.g. confidence/credible interval) and measures of statistical heterogeneity. If comparing groups, describe the direction of the effect.	
	20c	Present results of all investigations of possible causes of heterogeneity among study results.	Section 3.4 and 3.4 of Results
	20d	Present results of all sensitivity analyses conducted to assess the robustness of the synthesized results.	Section 3.3 of Results
Reporting biases	21	Present assessments of risk of bias due to missing results (arising from reporting biases) for each synthesis assessed.	Table S4
Certainty of evidence	22	Present assessments of certainty (or confidence) in the body of evidence for each outcome assessed.	Table S3
**DISCUSSION**			
Discussion	23a	Provide a general interpretation of the results in the context of other evidence.	Section 4.1-4.5 of Discussion
	23b	Discuss any limitations of the evidence included in the review.	The second limitation
	23c	Discuss any limitations of the review processes used.	The first limitation
	23d	Discuss implications of the results for practice, policy, and future research.	The last para. of Limitations
**OTHER INFORMATION**			
Registration and protocol	24a	Provide registration information for the review, including register name and registration number, or state that the review was not registered.	CRD42022340922
	24b	Indicate where the review protocol can be accessed, or state that a protocol was not prepared.	PROSPERO (york.ac.uk)
	24c	Describe and explain any amendments to information provided at registration or in the protocol.	We changed some details of the title to make it

Section and Topic	Item #	Checklist item	Location where item is reported
			better. Pro. Cheng did not participate in this study for personal issue, that’s why he was removed from the author list.
Support	25	Describe sources of financial or non-financial support for the review, and the role of the funders or sponsors in the review.	No support
Competing interests	26	Declare any competing interests of review authors.	No competing interests
Availability of data, code and other materials	27	Report which of the following are publicly available and where they can be found: template data collection forms; data extracted from included studies; data used for all analyses; analytic code; any other materials used in the review.	Data used for analyses can be obtained from the corresponding author upon making a reasonable request.

Table S2. Quality assessment checklist (10 out of 10 points)

**Categories**
**Category 1: Participants (5 points, 1 for each item)**
1) The patients were defined by DSM or ICD criteria and have suicide attempt or ideation
2) Controls were included based on the same inclusion criteria excluding the diagnosis of suicide
3) Sample size per group: ≥ 20 scores 1, one group ≥ 20 or both ≥ 10 scores 0.5
4) The baseline condition was defined as almost the same with task condition except for the executive control or decision making
5) All participants perform a preliminary experiment
**Category 2: Methods for image acquisition and analysis (3 points, 1 for each item)**
1) Head motion correction
2) The imaging techniques used were clearly described so that it could be reproduced
3) Important confounds (e.g. sex, age, year of education, severity of suicide) were controlled either by stratification or statistically
**Category 3: Results and conclusions (2 points, 1 for each item)**
1) Statistical results were corrected for multiple comparison
2) Conclusions were consistent with the results obtained and the limitations were discussed

*When criteria were partially met, 0.5 points were allocated

Table S3. Methodological details and quality score of the enrolled studies

Study	Software	STS	MRI scanner	Voxel size	Corrected level	Quality scores
Ai et al.[1]	SPM	MNI	3.0 T	3 × 3 × 3 mm	uncorrected p<0.001	8
Baek et al.[2]	SPM	MNI	3.0 T	2 × 2 × 2 mm	uncorrected p<0.001	7
Bomyea et al.[3]	AFNI	MNI	3.0 T	3.75 × 3.75 × 3 mm	uncorrected p<0.005, n>24	7.5
Dir et al.[4]	AFNI	Tal	3.0 T	2.5 × 2.5 × 2.5 mm	uncorrected p<0.01, n>201	9
Gorka et al. [5]	SPM	MNI	3.0 T	3.44 × 3.44 × 3.0 mm	uncorrected p < .005 , n > 171	9
Gifuni et al.[6]	AFNI	MNI	3.0T	3.1 × 3.1 × 4.0 mm	p-corrected <0.05 using AFNI's 3dClustsim algorithm	9
Ji et al.[6]	SPM	MNI	3.0 T	3.44 × 3.44 × 3.44 mm	uncorrected *p*<0.001	9
Jollant et al.[7]	SPM	MNI	1.5 T	3.75 × 3.75 × 3.75 mm	uncorrected p<0.001	8.5
Matthews et al.[8]	AFNI	Tal	3.0 T	2.5 × 2.5 × 2.5 mm	uncorrected p<0.05, cluster size>2048 mm3	7.5
Pan et al. (2011)[9]	SPM	MNI	3.0 T	3 × 3 × 3 mm	FDR corrected p<0.05	9.5
Pan et al. (2013)[10]	SPM	MNI	3.0 T	3 × 3 × 3 mm	FDR corrected p<0.05	9.5
Potvin et al.[11]	BrainVoyager QX	Tal	3.0 T	3.5 × 3.5 × 3.5 mm	uncorrected p<0.001, cluster size>343 mm^3	8.5
Richard-devantoy et al.[12]	SPM	MNI	3.0 T	3.5 × 3.5 × 3.5 mm	uncorrected p<0.001, n>10	9
Vanyukov et al.[13]	AFNI	MNI	3.0 T	3.5 × 3.5 × 3.5 mm	uncorrected p<0.001, n>22	8.5

Abbreviations: SPM, Statistical Parametric Mapping; AFNI, Analysis of Functional Neuro Images; MNI, Montreal Neurological Institute template; Tal, Talairach coordinates; STS, Stereotactic template space

Table S4. The use of medication for participants in original studies

Study	Medication
Ai et al.	Patients with suicide attempts and patient controls did not differ in medication use.
Baek et al.	There is no effect of current antidepressant treatment on risk or loss aversion in all depressed patients.
Bomyea et al.	The study did not included subjects who used psychotropic medication within four weeks prior to study entry.
Dir et al.	The study did not included subjects who use psychopharmacologic medications (other than psychostimulants) within the last 2 weeks.
Gorka et al.	The study did not included subjects who used any psychotropic medication.
Gifuni et al.	Patients with STB and patient controls, both receiving antidepressants, mood stabilizers, and low-dose neuroleptics. Sensitivity analysis controlling for medication status, psychiatric diagnoses, or head motion (average framewise displacement) did not change the pattern of neural activity related to group effects.
Ji et al.	The study did not included subjects who received any treatment in the preceding 6 months
Jollant et al.	Only three suicide attempters and one patient control took medication.
Matthews et al.	Patient and control groups were not different in prevalence of psychotropic medication use.
Pan et al. (2011)	Ten suicide attempters and six patient controls were treated with medication for depression.<br>Exploratory analyses did not reveal any significant relationships in either suicide attempters or patient controls between medications and activity in the neural regions showing between-group differences
Pan et al. (2013)	The study excluded those who used sedative or narcotic medication. In this study, ten suicide attemptes and seven patient controls were treated with medication for depression. Exploratory analyses did not show any significant relationships between medication status or load and activity during response inhibition in those regions showing abnormal activity.
Potvin et al.	Covariance analyses indicated that medication did not influence results.
Richard-devantoy et al.	Eight patient controls and 11 suicide attempters had never received an antidepressant medication before starting the study. For those who previously received an antidepressant, the washout period was 8.4 (S.D. = 2.7) days for patient controls and 6.2 (S.D. = 3.3) days for suicide attempters. None of them used fluoxetine and lithium previously.
Vanyukov et al.	The study ruled out potential confounders including medication exposure by different modeling approaches.

Table S5. Sensitivity analysis

Studies	Left anterior cingulate cortex	Right insula	Left precentral gyrus	Left hippocampus	left insula
**Decision-making**					
Baek et al.	Yes	Yes	NA	Yes	Yes
Dir et al.	Yes	Yes	NA	Yes	Yes
Gorka et al.	Yes	No	NA	Yes	Yes
Ji et al.	Yes	Yes	NA	Yes	Yes
Jollant et al.	Yes	Yes	NA	Yes	Yes
Pan et al. (2013)	Yes	Yes	NA	No	Yes
Potvin et al.	Yes	Yes	NA	Yes	Yes
Vanyukov et al.	Yes	Yes	NA	Yes	Yes
**Executive function**					
Ai et al.	Yes	NA	Yes	NA	NA
Bomyea et al.	Yes	NA	No	NA	NA
Gifuni et al.	No	NA	No	NA	NA
Matthews et al.	Yes	NA	Yes	NA	NA
Pan et al. (2011)	Yes	NA	No	NA	NA
Richard-devantoy et al.	Yes	NA	No	NA	NA

Table S6. Publication bias (Egger test)

Brain region	Bias	t	p
**Decision-making task**			
**STB > controls**			
R anterior cingulate cortex, BA 24	-1.87	-1.47	0.192
R insula, BA 48	-1.84	-1.48	0.191
**STB < controls**			
Left hippocampus, BA 20	-1.88	-1.63	0.154
L insula, BA 48	-1.23	-0.61	0.562

**Executive function task**			
**STB > controls**			
L cingulate cortex, BA 32	-1.30	-0.59	0.586
L postcentral gyrus, BA 4	-1.93	-1.03	0.360
**STB < controls**			
None			

STB: suicide thoughts and behaviors

References:

1. Ai H, van Tol MJ, Marsman JC et al. Differential relations of suicidality in depression to brain activation during emotional and executive processing. J Psychiatr Res, 2018;**105**:78-85.
2. Baek K, Kwon J, Chae JH et al. Heightened aversion to risk and loss in depressed patients with a suicide attempt history. Sci Rep, 2017;**7**:11228.
3. Bomyea J, Stout DM, Simmons AN. Attenuated prefrontal and temporal neural activity during working memory as a potential biomarker of suicidal ideation in veterans with PTSD. J Affect Disord, 2019;**257**:607-614.
4. Dir AL, Allebach CL, Hummer TA et al. Atypical Cortical Activation During Risky Decision Making in Disruptive Behavior Disordered Youths With Histories of Suicidal Ideation. Biol Psychiatry Cogn Neurosci Neuroimaging, 2020;**5**:510-519.
5. Gorka SM, Manzler CA, Jones EE, Smith RJ, Bryan CJ. Reward-related neural dysfunction in youth with a history of suicidal ideation: The importance of temporal predictability. Journal of Psychiatric Research, 2023;**158**:20-26.
6. Gifuni, A.J., et al., Decision-making and cognitive control in adolescent suicidal behaviors: a qualitative systematic review of the literature. Eur Child Adolesc Psychiatry, 2021. **30**: 1839-1855.

7. Ji X, Zhao J, Li H et al. From motivation, decision-making to action: An fMRI study on suicidal behavior in patients with major depressive disorder. J Psychiatr Res, 2021;**139**:14-24.
8. Jollant F, Lawrence NS, Olie E et al. Decreased activation of lateral orbitofrontal cortex during risky choices under uncertainty is associated with disadvantageous decision-making and suicidal behavior. Neuroimage, 2010;**51**:1275-1281.
9. Matthews S, Spadoni A, Knox K, Strigo I, Simmons A. Combat-exposed war veterans at risk for suicide show hyperactivation of prefrontal cortex and anterior cingulate during error processing. Psychosom Med, 2012;**74**:471-475.
10. Pan LA, Batezati-Alves SC, Almeida JR et al. Dissociable patterns of neural activity during response inhibition in depressed adolescents with and without suicidal behavior. J Am Acad Child Adolesc Psychiatry, 2011;**50**:602-611.e603.
11. Pan L, Segreti A, Almeida J et al. Preserved hippocampal function during learning in the context of risk in adolescent suicide attempt. Psychiatry Res, 2013;**211**:112-118.
12. Potvin S, Tikàsz A, Richard-Devantoy S, Lungu O, Dumais A. History of Suicide Attempt Is Associated with Reduced Medial Prefrontal Cortex Activity during Emotional Decision-Making among Men with Schizophrenia: An Exploratory fMRI Study. Schizophr Res Treatment, 2018;**2018**:9898654.
13. Richard-Devantoy S, Ding Y, Lepage M, Turecki G, Jollant F. Cognitive inhibition in depression and suicidal behavior: a neuroimaging study. Psychological Medicine, 2016;**46**:933-944.
14. Vanyukov PM, Szanto K, Hallquist MN et al. Paralimbic and lateral prefrontal encoding of reward value during intertemporal choice in attempted suicide. Psychological Medicine, 2015;**46**:381-391.
